# Pathogens protection against the action of disinfectants in multispecies biofilms

**DOI:** 10.3389/fmicb.2015.00705

**Published:** 2015-07-14

**Authors:** Pilar Sanchez-Vizuete, Belen Orgaz, Stéphane Aymerich, Dominique Le Coq, Romain Briandet

**Affiliations:** ^1^INRA, UMR1319 MICALIS, Jouy-en-JosasFrance; ^2^AgroParisTech, UMR MICALIS, Jouy-en-JosasFrance; ^3^Department of Nutrition, Food Science and Technology, Faculty of Veterinary, Complutense University de MadridMadrid, Spain; ^4^CNRS, Jouy-en-JosasFrance

**Keywords:** multispecies biofilm, disinfectants, bacterial pathogens, protection, interspecies interactions

## Abstract

Biofilms constitute the prevalent way of life for microorganisms in both natural and man-made environments. Biofilm-dwelling cells display greater tolerance to antimicrobial agents than those that are free-living, and the mechanisms by which this occurs have been investigated extensively using single-strain axenic models. However, there is growing evidence that interspecies interactions may profoundly alter the response of the community to such toxic exposure. In this paper, we propose an overview of the studies dealing with multispecies biofilms resistance to biocides, with particular reference to the protection of pathogenic species by resident surface flora when subjected to disinfectants treatments. The mechanisms involved in such protection include interspecies signaling, interference between biocides molecules and public goods in the matrix, or the physiology and genetic plasticity associated with a structural spatial arrangement. After describing these different mechanisms, we will discuss the experimental methods available for their analysis in the context of complex multispecies biofilms.

## Introduction

In nature, microorganisms are commonly found living associated to surfaces and enclosed in self-generated extracellular polymers that maintain them together forming biofilms (Costerton et al., 1995). These organized communities are essential to ensure an ecological equilibrium as the inhabitants of biofilms are characterized by their survival under stressful conditions such as desiccation or nutrient starvation and their participation in the global biogeochemical cycle (Burmølle et al., 2012). Biofilms are also found in man-made environments, where they may be related to nosocomial infections, food spoilage, and damage to industrial pipelines (Hall-Stoodley et al., 2004; Bridier et al., 2011a; Flemming, 2011a). After more than 30 years of intensive research, extensive knowledge has been accumulated on the mechanisms that govern this multicellular behavior, such as the production of matrix polymers, cell–cell communication, or the generation of multiple cell types within the biostructure (Stewart, 2002; Høiby et al., 2010; Bridier et al., 2011a). Most of those pioneer studies were performed on single-strain biofilms, probably because of the experimental limitations associated with more complex communities. However, simple laboratory models are hardly representative of natural biofilms where multispecies communities are by far the most predominant (Hall-Stoodley et al., 2004). The presence of different partners in the biofilm matrix renders both the structure and function of the community more complex and mechanisms other than those considered in single-strain biofilms need to be considered.

Interspecies interactions can drive ecological advantages in a biofilm. For example, the establishment of a mixed biofilm favors the uptake by *Pseudomonas* sp. of the waste substances secreted by *Burkholderia* sp. in the presence of the pollutant chlorobiphenyl (Nielsen et al., 2000). Likewise, the spatial organization and stratification of incompatible bacteria, such as aerobic nitrifiers and anaerobic denitrifiers, allows their co-metabolism and the degradation of toxic compounds (Terada et al., 2003). The anthropocentric negative impact of interactions between species is reflected in biofilms related to chronic infections. The colonization by multiple pathogenic species of native tissues such as the lungs of cystic fibrosis patients, chronic wounds, or the urinary tract frequently induces more severe and recalcitrant infections (Wolcott et al., 2013). For instance, co-infection by *Pseudomonas aeruginosa* and *Staphylococcus aureus* delays wound healing and trigger host inflammatory response (Seth et al., 2012; Pastar et al., 2013). Similarly *S. aureus* virulence is induced in the presence of *P. aeruginosa* or the fungus *Candida albicans* (Hendricks et al., 2001; Peters et al., 2010) as well as *P. aeruginosa* exhibited enhanced virulence in a *Drosophila* model when it was co-inoculated with Gram-positive bacteria (Korgaonkar et al., 2013). Moreover, recent works have reflected a growing concern about the increasing resistance of pathogens to antibiotics observed in multispecies communities (Adam et al., 2002; Al-Bakri et al., 2005; Luppens et al., 2008; Harriott and Noverr, 2009; Lopes et al., 2012; Lee et al., 2014).

Multispecies interactions are also involved in the persistence of pathogens on inert surfaces in medical or industrial environments. In such cases, the biocontamination of equipment is associated with nosocomial and foodborne infections despite frequent and intensive cleaning and disinfection procedures (Mack et al., 2006; Shirtliff and Leid, 2009; Bridier et al., 2015). Unlike antibiotics, which usually have a specific target, disinfectants are multi-target agents (e.g., cell wall, proteins, DNA, and RNA) whose actions typically cause disruption of the bacterial membrane (Maillard, 2002). Although these biocides are highly effective on planktonic bacteria, their efficacy relative to spatially organized biofilms is open to question in light of some published reports (Russell, 1999; Bridier et al., 2011a; Davin-Regli and Pagès, 2012; Abdallah et al., 2014). The tolerance of biofilm-dwelling cells to disinfectants is attributed to multiple factors, often operating in concert, and which include the presence of extracellular polymers that hamper their diffusion/reaction, and differences in physiological status depending on the biofilm stratum (Stewart and Franklin, 2008; Bridier et al., 2011a). There is also increasing evidence that interspecies interactions within the matrix further increase the tolerance against disinfectants observed in single-strain biofilms (Burmølle et al., 2006; Bridier et al., 2012; Schwering et al., 2013; Wang et al., 2013). However, the specific mechanisms underlying this tolerance are still poorly understood, and their clarification is difficult due to the complexity and heterogeneities of these biostructures.

Some of the mechanisms by which biofilms cells are resistant to antibiotics are likewise behind the resistance to disinfectants. This review therefore focuses on the mechanisms involved in the tolerance and resistance to disinfectants of multispecies biofilms, with particular attention to the protection of pathogenic species. The experimental methods available for the study of spatially organized multispecies communities, and their response to biocides, will also be reviewed.

## Do Mixed-Species Biofilms Tolerate the Action of Biocides Better than their Single-Strain Counterparts?

It is becoming increasingly obvious that social behavior within a mixed community confers bacterial tolerance to environmental stresses, including the action of disinfectants that until now has been largely underestimated. **Table 1** presents a great number of studies showing an increased resistance to disinfectants in multispecies biofilms. For example, four species isolated from a marine alga formed a multispecies biofilm with increased biomass and a eightfold enhancement in its tolerance to hydrogen peroxide when compared to its single-strain counterparts (Burmølle et al., 2006). Similarly, the association in a mixed biofilm of *Bacillus cereus* and *Pseudomonas fluorescens* two species frequently isolated on surfaces in food processing industries, led to a remarkable increase in their tolerance to two frequently used disinfectants, chloride dioxide and glutaraldehyde (Lindsay et al., 2002; Simões et al., 2009). In some reports, a “public good” produced by one species has been observed to offer protection for the whole population. One example is the curli-producer *Escherichia coli* that was found to protect *Salmonella Typhimurium* in a dual-species biofilms when subjected to chlorine (Wang et al., 2013).

**Table 1:** Species associations leading to increased biocidal resistance in biofilms as determined by studies so far.

Biocide	Species	Conditions for biofilm formation	Reference
Chloride dioxide	*B. cereus, P. fluorescens*	Flow cell chamber	Lindsay et al. (2002)
Glutaraldehyde	*B. cereus*, *P. fluorescens*	Stainless steel coupons	Simões et al. (2009)
Essential oils	*P. putida*, *S. enterica*, *L. monocytogenes*	Stainless steel coupons	Chorianopoulos et al. (2008)
Essential oils	*S. aureus*, *E. coli*	Polypropylene coupons	Millezi et al. (2012)
Peracetic acid	*Listeria innocua*, *P. aeruginosa*	Stainless steel coupons	Bourion and Cerf (1996)
Peracetic acid Ortho-phthalaldehyde acid	*B. subtilis*, *S. aureus*	Microtiter plates	Bridier et al. (2012), Sanchez-Vizuete et al. (2015)
Chlorhexidine Hydrogen peroxide	*S. mutants*, *V. parvula*	Microtiter plates	Kara et al. (2006), Luppens et al. (2008)
Chlorine	*Kocuria* sp., *Brevibacterium linens*, *S. sciuri*	Stainless steel coupons	Leriche et al. (2003)
Chlorine	*9 drinking water system flora, E. coli*, *P. aeruginosa* *Stenotrophomonas maltophilia*, *E. cloacae*	Calgary biofilm device	Schwering et al. (2013)
Betadine	*P. putida*, *Vogesella indigofera*	Chemostat reactor	Whiteley et al. (2001)
Hydrogen peroxide	*Methylobacterium phyllosphaerae, Shewanella japonica* *Dokdonia donghaensis, Acinetobacter lwoffii*	Microtiter plates	Burmølle et al. (2006)
Benzalkonium chloride	*L. monocytogenes*, *P. putida*	Stainless steel and polypropylene coupons	Saá Ibusquiza et al. (2012)
Chlorine	*E. coli*, *S. Typhimurium*	Microtiter plates	Wang et al. (2013)
Chlorine	*S. Typhimurium*, *P. fluorescens*	Polycarbonate coupons	Leriche and Carpentier (1995)
Benzalkonium chloride Peracetic acid	*L. monocytogenes*, *Lb. plantarum*	Microtiter plates	van der Veen and Abee (2011)
Isothiazolone	*Alcaligenes denitrificans*, Pseudomonas *alcaligenes* *S. maltophilia*, *Fusarium oxysporum,* *Flavobacterium indologenes Fusarium solani,* *Rhodotorula glutinis*	Flow cell system	Elvers et al. (2002)
Benzalkonium chloride	*P. putida*, *L. monocytogenes*	Stainless steel coupons	Giaouris et al. (2013)
Chlorhexidine	*S. mutants*, *S. aureus*, *P. aeruginosa*	Titanium disk	Baffone et al. (2011)
Carvacrol Chlorhexidine	*S. mutans*, *Porphyromonas gingivalis* *Fusobacterium nucleatum*	Titanium disk	Ciandrini et al. (2014)
SDS	*Klebsiella pneumoniae*, *P. aeruginosa* *P. fluorescens*	Flow cell system	Lee et al. (2014)
Chlorine	*P. aeruginosa*, *B. cepacia*	Chemostat reactor	Behnke et al. (2011)
Sodium hypochlorite	*A. calcoaceticus*, *B. cepacia,* *Methylobacterium* sp. *Mycobacterium mucogenicum, Sphingomonas capsulata,* *Staphylococcus* sp.	Microtiter plates	Chaves Simões et al. (2010)

One of the most worrying issues raised by recent findings is that resident surface flora have been shown to protect pathogens from biocide action in different situations. In one example, the presence of *Veillonella parvula* in an oral biofilm enabled a 50% increase in the survival rate of *Streptococcus mutans* when subjected to five different antimicrobial agents (Kara et al., 2006; Luppens et al., 2008); in other cases of multispecies biofilms, *Lactobacillus plantarum* protected *Listeria monocytogenes* from the action of benzalkonium chloride and peracetic acid (van der Veen and Abee, 2011), while a biofilm formed by nine environmental species protected different pathogens (*E. coli*, *Enterobacter cloacae*, *P. aeruginosa)* against the action of chlorine (Schwering et al., 2013). The importance of resident flora in foodborne or nosocomial infections is often neglected because these strains are generally non-virulent. However, they may be particularly persistent due to adaptation mechanisms that are associated with their frequent exposure to biocides, and thus provide shelter for pathogenic strains. For instance, a study showed that a *Bacillus subtilis* strain isolated from an endoscope washer-disinfector, which was particularly resistant to the high concentrations of oxidative disinfectants used daily in these devices, was able to protect *S. aureus* from the action of peracetic acid within a multispecies biofilm (Bridier et al., 2012). Similarly, it was demonstrated in a recent work that resident flora from lettuce increases *S. Typhimurium* resistance to UV-C irradiation in this habitat (Jahid et al., 2015).

These telling examples should not lead us to believe that bacterial protection in multispecies biofilms is a universal trait. Thus the food-borne pathogen *L. monocytogenes* can be protected from biocide action in a mixed biofilm by *Lb. plantarum* (van der Veen and Abee, 2011), but not by *Salmonella enterica* or *P. putida* (Chorianopoulos et al., 2008; Kostaki et al., 2012). Likewise, the complex biofilms formed by *S. aureus*, *P. aeruginosa*, and *C. albicans* were shown to be more susceptible to some antimicrobials than their single-strain homologous counterparts (Kart et al., 2014). *Enterococcus faecalis* was also found more susceptible to sodium hypochlorite when cultured with two oral bacteria (Yap et al., 2014). In light of these studies, the evaluation of specific interspecies interactions, either leading to higher or lower susceptibility to disinfectants, becomes of extreme importance in order to establish new strategies against pathogens persistence.

## Mechanisms Involved in Interspecies Protection

Some of the mechanisms involved in the tolerance of axenic biofilm-dwelling cells to disinfectants action can be applied to multispecies communities. However, in most situations the specific interactions between different species make it necessary to consider other mechanisms that are not observed in single-strain biofilms.

### The Biofilm Matrix as an Interspecies Public Good

Biofilm cells produce extracellular polymeric substances that hold them together and favor the three-dimensional spatial arrangement (Branda et al., 2005). While the biofilm matrix mostly contains polysaccharides, proteins, lipids, and DNA, its composition can differ markedly depending on environmental conditions, the species, and even between different strains of the same species (Flemming and Wingender, 2010; Bridier et al., 2012; Combrouse et al., 2013). Although biocides can gain direct access to their microbial targets in planktonic cultures, they may encounter diffusion-reaction limitations through the matrix of polymers so that they hardly reach the deepest layers of the biofilm in their active form (Stewart et al., 2001; Jang et al., 2006; Bridier et al., 2011b). Multispecies biofilms are often associated with increased matrix production and because of the complexity of its biochemical nature this may exacerbate such diffusion-reaction limitations (Skillman et al., 1998; Sutherland, 2001; Andersson et al., 2011).

The protective function of the matrix may be associated with specific components produced by one species that benefit the whole population (Flemming, 2011b). This is the case of enzymes secreted in the matrix by one strain that may alter the reactivity of the biocide; e.g., secretion of a specific hydrolase by *P. aeruginosa* was found to confer tolerance to SDS on a mixed community (Lee et al., 2014). Other matrix components with protective functions are amyloids, a specific class of highly aggregated proteins associated with different bacterial functions such as adhesion, cohesion, and host interactions (Pawar et al., 2005; Tükel et al., 2010; Blanco et al., 2012). The best described biofilm-associated amyloids are TasA in *B. subtilis*, FapC in *Pseudomonas* sp., and curli in *E. coli* or *Salmonella* sp. (Chapman et al., 2008; Romero et al., 2010; Dueholm et al., 2013). Amyloids have also been detected in natural multispecies biofilms, such as the communities formed by *S. enterica* and *E. coli,* two species able to cooperate and share curli subunits *in vivo* in the context of a process called cross-seeding (Zhou et al., 2012). Interestingly, a significant increase in the tolerance of *E. coli* cells to biocides was observed in a mixed biofilm when associated with a curli-producing *S. enterica* strain, but not with a non-producer. Symmetrically, the biocidal tolerance of an *S. enterica* non-producing strain was enhanced when it grew with a strain of *E. coli* producing curli (Wang et al., 2013). The effect of protection observed is probably due to the sharing of curli subunits whose polymerization may be accelerated by preformed amyloid aggregates as it has been shown in yeasts (Glover et al., 1997).

The BslA amphiphilic protein produced by *B. subtilis* has been shown to form a protective coating at the interface between a macrocolony on agar and air. This hydrophobic coating prevents the penetration of biocides and protects the matrix inhabitants (Epstein et al., 2011; Kobayashi and Iwano, 2012). This “molecular umbrella” is a typical public good of the matrix that may benefit other species in the community. As well as these specific protective components, sharing the matrix with other species can trigger an increase in the synthesis of a precise polymer or in the number of producing cells, and hence the abundance of biocide-interfering organic material (Leriche and Carpentier, 1995; Lindsay et al., 2002; Simões et al., 2009). This is the case with the *B. subtilis* TasA amyloid matrix protein that is mostly overproduced in the presence of other strains from the *Bacillus* genus (Shank et al., 2011). Coaggregation between bacteria of different species can promote matrix synthesis, the overall biofilm population and tolerance to biocides, e.g., the oral pathogen *S. mutans* was found to coaggregate with the early colonizer *V. parvula* and this resulted in a multispecies biofilm that produced more matrix and was more tolerant to chlorhexidine and five other biocides than the corresponding axenic biofilms (Kara et al., 2006; Luppens et al., 2008). Similarly, the coaggregation of six strains isolated from a drinking water system was also suggested to explain the high tolerance to sodium hypochlorite of the multispecies consortia (Chaves Simões et al., 2010). Another mechanism is metabolic cross-feeding between species that can promote the growth of biofilm-dwelling cells and enhance their survival when challenged by biocides (Kara et al., 2006; Ramsey et al., 2011; Stacy et al., 2014).

Populations of cells over-expressing biocide-interfering components can also emerge in the community through the selection of specific mutants (Morris et al., 1996; Boles et al., 2004; Römling, 2005; Uhlich et al., 2006; Starkey et al., 2009; Singh et al., 2010). This emergence of genetic variants may be stimulated under multispecies conditions. This was the case of *P. putida* variants evolving phenotypically distinct morphologies that resulted in a more stable and productive community in the presence of a strain of *Acinetobacter* sp. (Hansen et al., 2007). A recent study revealed a synergistic genetic diversification of the model strain *P. putida* KT2440 in the presence of an environmental isolate of *P. putida*, but not in single-strain biofilms (Bridier et al., under revision).

### Spatially Driven Cellular Physiology in Mixed Communities

Microorganisms are not randomly organized within a multispecies biofilm, but follow a pattern that contributes to the fitness of the whole community (Marsh and Bradshaw, 1995; Rickard et al., 2003; Robinson et al., 2010), e.g., species are organized in layers, clusters, or are well-mixed (Elias and Banin, 2012). This spatial organization partially determines bacterial survival when the biofilm is exposed to toxic compounds (Simões et al., 2009). This depends to a great extent on interactions between the species and their local micro-environments in the matrix with respect to nutrient, oxygen, and metabolite gradients (Stewart and Franklin, 2008). In a mixed biofilm, matrix reinforcement and competition for resources can intensify the slope of these gradients, and hence the physiological diversification of the population, including tolerant slow-growth cells. Oxygen depletion in spatially organized multispecies biofilms was suggested as an explanation for the protection of *Staphylococcus sciuri* by *Kocuria* sp. when exposed to chlorine (Leriche et al., 2003). The structured association of *Burkholderia cepacia* and *P. aerugino*sa and their related cell physiologies also led to a higher rate of survival following exposure to chlorine (Behnke et al., 2011). A specific sub-population of cells described as persisters corresponds to phenotypic variants that are present in small proportions in the biofilm but are highly tolerant to killing by biocides (Lewis, 2010). As yet, the generation of persister cells in multispecies biofilms has been little investigated but it is known that they emerge under stressful situations such as nutrient limitation or oxidative stress (Wang and Wood, 2011). It has been demonstrated that the siderophore pyocyanin is secreted by *P. aeruginosa* in order to generate oxidative stress and thus to compete with other bacteria (Tomlin et al., 2001). Thus, exogenous pyocyanin has been shown to trigger the appearance of a sub-population of persister cells in *Acinetobacter baumannii*, an emerging pathogen isolated from the same sites of infection as *P. aeruginosa* and able to form mixed biofilms with it (Bhargava et al., 2014).

### Interspecies Communication

Quorum sensing (QS) signals, known as autoinducers (AI), can be used for intra-species cell-to-cell communication, as is the case of acyl-homoserine lactones (AHLs) in Gram-negative microorganisms, and modified oligopeptides in Gram-positive microorganisms (Parsek and Greenberg, 2000; Miller and Bassler, 2001). They induce coordinated responses for the development of genetic competence, the regulation of virulence and biofilm formation (Jayaraman and Wood, 2008). These cell-to-cell communication mechanisms may play a role in governing specific gene expression in order to modulate the biocidal resistance of biofilms (Hassett et al., 1999). Autoinducer-2 (AI-2) is considered to be a universal language molecule that is well suited to interspecies communication between microorganisms (West et al., 2012; Pereira et al., 2013). AI-2 has been detected and produced by a variety of microorganisms isolated from chronic wounds (Rickard et al., 2010). One species may therefore interfere with the signaling pathway of other species in a biofilm, either stimulating, inhibiting, or inactivating QS signals (Bauer and Robinson, 2002; Zhang and Dong, 2004; Elias and Banin, 2012; Rendueles and Ghigo, 2012). These interferences may alter gene expression or be more than a “simple message” directly affecting the physiology of the co-habitants (Schertzer et al., 2009). It has been shown that the biofilm formation and antimicrobial resistance of a mixed community formed by the opportunistic pathogen *Moraxella catarrhalis* and *Haemophilus influenzae* is promoted by the A1-2 QS signal produced by *H. influenzae* (Armbruster et al., 2010). Signaling within a dual-species oral bacteria community has also been reported (Egland et al., 2004). These authors showed that *Veillonella atypical* produced a signal that caused *Streptococcus gordonii* to increase the expression of the gene coding for an α-amylase.

The ability of certain microorganisms to produce enzymes that interfere with the communication system of other species is considered as a primary defense mechanism of bacteria (Chen et al., 2013). For instance, some species of *Bacillus* produce AHL-lactonases that inhibit the formation of biofilms of other pathogenic species (Dong et al., 2001; Wang et al., 2007). QS molecules may also exhibit antimicrobial properties, as has been described for the auto-inducer CAI-1 produced by *Vibrio cholerae.* This QS signal exerts a dual effect on the inhibition of *P. aeruginosa*, in a concentration-dependent manner; whereas at low concentrations it was seen to inhibit *P. aeruginosa* QS, at higher concentrations this AI caused pore formation in *Pseudomonas* membrane, leading to cell death (Ganin et al., 2012). Under iron-limited conditions, the transcription of iron-regulated genes in *P. aeruginosa* was decreased in the presence of *S. aureus* (Mashburn et al., 2005). QS molecules produced by *P. aeruginosa* probably induce the lysis of *S. aureus* and its use as an iron source. By contrast, other QS signals may act as iron chelating molecules (Bredenbruch et al., 2006).

Alongside the classic QS mediators, recent studies have highlighted a signaling activity for the exopolysaccharides produced by the *B. subtilis eps* operon. This polymer is recognized by the extracellular domain of a tyrosine kinase which activates its own synthetic pathway (Elsholz et al., 2014). Similarly, in *P. aeruginosa,* it has been demonstrated that the Ps1 polymer stimulates matrix production in neighboring cells *via* c-di-GMP activation, although the precise mechanism remains unknown (Irie et al., 2012).

### Genetic Plasticity in Multispecies Biofilms

The intercellular space of a biofilm offers an excellent reservoir of genetic material that can be exchanged between species. The physical proximity and presence of extracellular DNA (eDNA) in the matrix facilitates horizontal gene transfer (HGT) between species (Christensen et al., 1998; Hausner and Wuertz, 1999). It has been demonstrated that *S. epidermis* produced more eDNA when in a mixed biofilm with *C. albicans* leading to an increased biofilm biovolume and an enhanced infection in a *in vivo* model (Pammi et al., 2013). HGT is a prevalent driving mechanism for bacteria, enabling them to acquire new genetic material that provides antimicrobial resistance and other functionalities which can promote their persistence in natural environments (de la Cruz and Davies, 2000; Barlow, 2009; Wiedenbeck and Cohan, 2011). In *Vibrio cholera* it has been demonstrated that HGT can be induced in response to AI derived from other *Vibrio* species in multispecies biofilms (Antonova and Hammer, 2011). Genetic determinants for biofilm formation can also be transferred between *E. coli* and *S. enterica*, as has been hypothesized to occur in a biofilm formed by curli-producing and non-producing strains (Wang et al., 2013).

Resistant mutants can also emerge spontaneously in the population under stressful conditions such as exposure to antimicrobial agents (Cantón and Morosini, 2011). In mixed biofilms, interactions and competition between species can enhance the emergence of genetic variants, as demonstrated for *P. aeruginosa* in the presence of *C. albicans* (Trejo-Hernández et al., 2014).

## Experimental Methods to Study Multispecies Biofilms and their Response to the Action of Biocides

**FIGURE 1:**
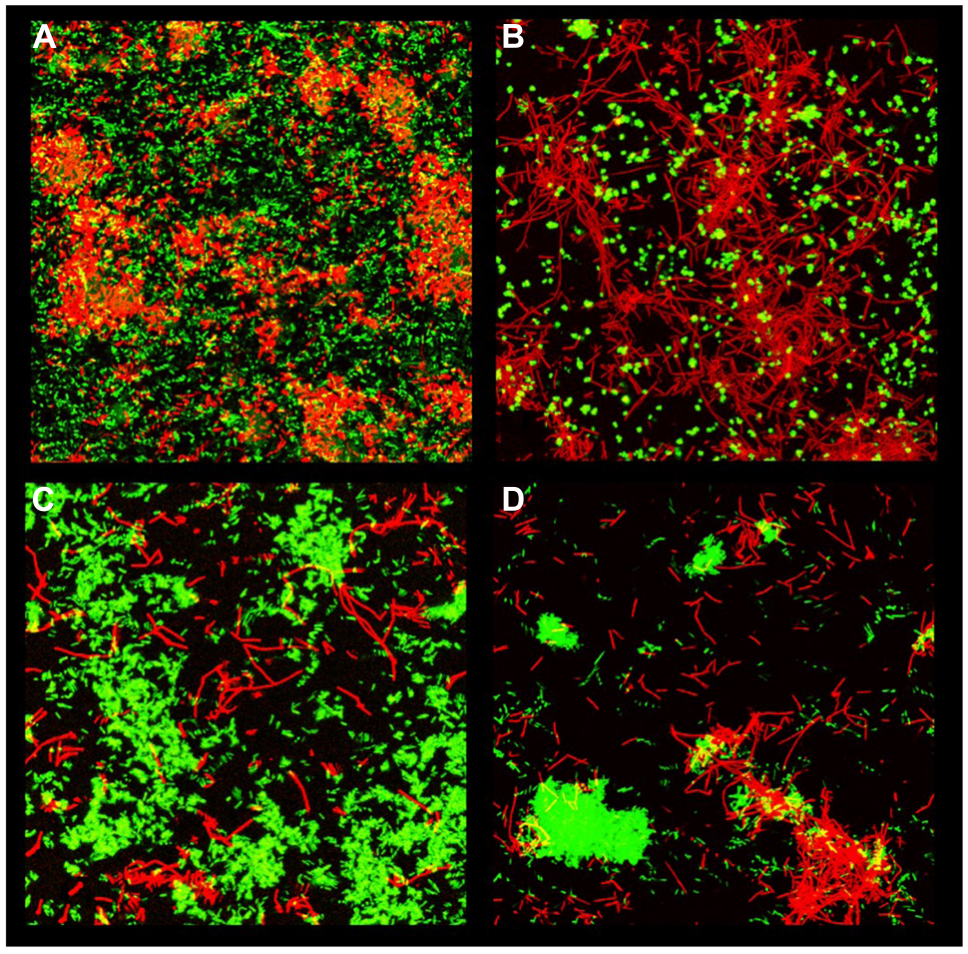
**Spatial organization in mixed-species biofilms.** *B. subtilis* NDmed mCherry (red) displays a specific distribution when grown with different pathogenic partners (green). *B. subtilis* with **(A)** *S. enterica* GFP **(B)** *S. aureus* GFP, **(C)** *E. coli* K12 GFP, or **(D)** *E. coli* SS2 GPF.

The establishment of a multispecies biofilm is a complex biological process that involves interspecies interactions (cooperation, antagonism, etc.). Re-creating these driving interactions in the laboratory is one of the most difficult challenges that researchers must face when growing multispecies biofilms. Most published studies have involved two or three species because of the problems encountered in setting up a repeatable biostructure. Strains and growth conditions (e.g., temperature, culture media, and biofilm set-up) must be chosen and controlled with particular care, otherwise the results obtained can be distinct. The **Figure 1** shows different spatial interactions between the hospital isolate of *B. subtilis* NDmed and four different pathogens species. Another important choice is the disinfectant agent used to treat the mixed-biofilm. For example, a mixed biofilm of *P. fluorescens* and *B. cereus* led to an increase in the tolerance of both species to a surfactant and an aldehyde when cultivated in a rotating stainless steel device for 7 days (Simões et al., 2009); however, when co-cultured in a flow system for 16 h, *B. cereus* proved to be more susceptible to the oxidant agent chlorine than in an axenic biofilm (Lindsay et al., 2002). Although the techniques available to study biofilms have evolved significantly during recent decades (confocal laser microscopy, fluorescent reporters, micro-electrodes, etc.), the analysis of multispecies biofilms still remains a technical challenge due to the lack of methods adapted to complex communities and to the difficulty of preserving certain fundamentals traits in these complex samples.

### Visualization of the Spatial Organization of Species in Multispecies Biofilms

Confocal laser scanning microscopy (CLSM) coupled with specific fluorescent labeling has emerged as a non-invasive technique that is widely used for the *in situ* observation of the structure and reactivity of biofilms. Nucleic acid stains, such as Syto9 or SYBR Green are widely used to label individual cells and visualize biofilm architecture (Bridier et al., 2010). However, in a multispecies context, this approach cannot discriminate between each species in the structure. Fluorescent *in situ* hybridization (FISH) has appeared as a powerful tool allowing the visualization of both laboratory and environmental multispecies biofilms (Thurnheer et al., 2004; Amann and Fuchs, 2008). Fluorescent DNA probes specifically designed for each species and labeled with a fluorophore of a given color hybridize to bacterial ribosomal RNA, even if cells are in a “dormant” state (Baudart et al., 2005; Servais et al., 2009). Limitations of this technique in terms of probes diffusion within the biofilm, penetration into the cell and binding to nucleic acids (Amann and Fuchs, 2008; Almstrand et al., 2013) have been overcome with the use of peptide nucleic acid (PNA) (Stender et al., 2002; Cerqueira et al., 2008; Almeida et al., 2009). Coupled with CLSM, this method enables the study of the composition of multispecies communities and their spatial organization without drastically affecting their biological structure (Dige et al., 2009; Malic et al., 2009; Almeida et al., 2011). As an alternative to PNA-FISH and when antibodies are available, immunofluorescence can be used to visualize one or two species of interest within a community (Guiamet and Gaylarde, 1996; Hausner et al., 2000; Chalmers et al., 2008). At the single-cell level, techniques such as microautoradiography (MAR), Raman spectroscopy, and secondary ion mass spectrometry (SIMS), that use isotope labeling to detect and quantify metabolic activities, have been applied to complex communities in combination with FISH in order to obtain information not only about the community composition but also the metabolic state or the molecular composition (Lee et al., 1999; Orphan et al., 2001; Kindaichi et al., 2004; Nielsen and Nielsen, 2005; Huang et al., 2007; Wagner, 2009; Musat et al., 2012).

When dynamic information is required, a set of mutant strains expressing fluorescent proteins can be used simultaneously in a multispecies biofilm, i.e., one strain expressing the green fluorescent protein (GFP), the other strain expressing the red fluorescent protein (RFP), (Rao et al., 2005; Moons et al., 2006; Ma and Bryers, 2010). *In situ* 4D confocal imaging enables recovery of the spatio-temporal patterns of colonization of each species within the biostructure. Although it is theoretically possible to monitor more than four or five types of cells in a biofilm using this approach, technical limitations usually restrict the acquisitions to two or three cell types in the same sample (Klausen et al., 2003; Bridier et al., 2014). Fluorescent proteins are also widely used to reveal the expression of specific genes in the biofilm with single cell resolution, as well as protein localization (Christensen et al., 1998; Ito et al., 2009; Wei et al., 2011; Moormeier et al., 2013). However, the use of such fluorescent reporter technologies is limited to strains that can be genetically manipulated and to the intensity of the fluorescence they emit, which in turn is dependent on the local pH and oxygen content (Hansen et al., 2001).

### Quantification of the Action of Biocides in a Multispecies Biofilm

Quantifying the action of a biocide on a biofilm population can be achieved using global invasive approaches such as CFU counting, the Calgary Biofilm Device, the crystal violet assay, or the respiration assay with TTC (Ceri et al., 1999; Ren et al., 2014; Sabaeifard et al., 2014). CFU counting on different selective agar media can estimate the cultivable fraction of each species in a sample (Seth et al., 2012; Giaouris et al., 2013; Schwering et al., 2013); however, not all bacterial species are able to grow in laboratory [viable but non-cultivable (VBNC) subpopulation] or need interspecies interactions to grow (Trevors, 2011; Li et al., 2014). Besides, the complete detachment of cells from the surface and the effective disruption and resuspension of biofilm aggregates are a concern when applying these culture-based approaches. Real-time quantitative PCR (qPCR) has emerged as a successful molecular tool for the identification and quantification of specific microorganisms in multispecies communities (Maciel et al., 2011; Pathak et al., 2012). This technique allows discrimination between live and dead cells by the combination of specific amplification of rRNA regions and the use of propidium monoazide (PMA) able to penetrate compromised or damaged membranes, intercalate DNA, and prevent its amplification (Nocker et al., 2006). This method was recently applied to the study of antimicrobial resistance in multispecies biofilms (Alvarez et al., 2013; Yasunaga et al., 2013; Kucera et al., 2014; Sánchez et al., 2014). Although it was found to be relatively efficient, molecular analysis require expensive preparation and the protocols need to be adapted to each condition because of the considerable complexity of multispecies biofilms. Recent studies have also demonstrated that qPCR-PMA tends to overestimate the fraction of live cells (Løvdal et al., 2011; Slimani et al., 2012; Gensberger et al., 2013). Flow cytometry can also be applied to quantify viability of different bacterial species after resuspension of multispecies biofilms. As an example, the viability of *P. aeruginosa*, *B. cepacia*, and *S. aureus* in a mixed culture was quantified by means of fluorescence detection using multifluorescent labeling with antibody, lectins, SYBR Green and propidium iodide (Rüger et al., 2014). This method has also been applied to *P. aeruginosa* axenic biofilms in order to separate active and dormant cell populations and compare their phenotypes and resistance to various antimicrobial agents (Kim et al., 2009).

The techniques presented so far are performed on detached and resuspended biofilms, losing thus the spatial information on the community. Some microscopic approaches are able to combine viability status at single cell resolution with other information such as the species localization or function. LIVE/DEAD staining and esterase activity dyes have been applied successfully for the real-time visualization of cell inactivation in biofilms (Takenaka et al., 2008; Harmsen et al., 2010; Bridier et al., 2011b; Løvdal et al., 2011). One interesting approach to decipher biocidal limitations in multispecies biofilms is to combine such dyes with species-specific labeling or fluorescent lectins (Neu et al., 2001).

## Concluding Remarks

Today, the non-specific and disproportionate utilization of biocides is causing major problems of environmental pollution (Martinez, 2009; Moellering, 2012). Now that society begins to be aware of increasing bacterial resistance to antibiotics, a growing number of studies have reported cross-resistance events between different types of antimicrobials, such as disinfectants and antibiotics (Gilbert et al., 2002; Davin-Regli and Pagès, 2012). One process giving rise to the tolerance bacteria to chemical disinfectants, and which has been largely underestimated in recent years, is interspecies bacterial interactions in spatially organized biofilms. One significant concern regarding these biological associations is the increase of pathogens persistence that is favored by the protection of resident flora. The studies reviewed in this paper highlight the pressing need to gain a clearer understanding of the specific mechanisms associated with these protective effects. Although the spatial organization of a mixed community is fundamental to its response to antimicrobials, little use is still made of visualization techniques such as PNA-FISH or real-time CLSM. New standardized protocols need to be established in order to decipher the associated mechanisms and support the development of specific control strategies with respect to multispecies biofilms.

## Conflict of Interest Statement

The authors declare that the research was conducted in the absence of any commercial or financial relationships that could be construed as a potential conflict of interest.
